# Current Progress and Outlook for Agrimonolide: A Promising Bioactive Compound from *Agrimonia pilosa* Ledeb.

**DOI:** 10.3390/ph16020150

**Published:** 2023-01-19

**Authors:** Ting Huang, Chun-Cao Zhao, Man Xue, Yun-Feng Cao, Liang-Kang Chen, Jian-Xing Chen, Yi-Jie Sun, Jia Zeng

**Affiliations:** 1 Shanghai Institute for Biomedical and Pharmaceutical Technologies, NHC Key Lab of Reproduction Regulation, Shanghai Engineer and Technology Research Center of Reproductive Health Drug and Devices, Shanghai 200032, China; 2 Huadong Hospital Affiliated to Fudan University, Shanghai 200040, China

**Keywords:** agrimonolide, *Agrimonia pilosa* Ledeb., pharmacological effect, safety

## Abstract

Agrimonolide (AM), which is a derivative of isocoumarins, is found mainly in the herb *Agrimonia pilosa* Ledeb. This compound is highly lipophilic and readily crosses the blood–brain barrier. In recent years, interest has grown in the use of AM as a multitarget natural treatment for various diseases, such as cancer, inflammation, hepatic injury, myocardial damage, and diabetes mellitus. The potential mechanisms of these pharmacological effects have been clarified at cellular and molecular levels. AM shows no cytotoxicity over a range of concentrations in different types of cells, providing evidence for its good safety profile in vitro. These findings indicate that AM is a promising medicinal agent. However, most studies on AM’s pharmacological activities, mechanisms of action, and safety lack substantial animal or human data. Additionally, the pharmacokinetics, metabolism, and disposition of this compound have received little attention. This review highlights the status of current information regarding the sources, properties, pharmacological effects, and safety of AM. Furthermore, potential strategies to resolve problematic issues identified in previous studies are fully discussed. This summary and analysis of the research progress of AM may inspire deeper investigations and more extensive applications of AM in the future.

## 1. Introduction

Agrimonolide (AM), also known as agrimolide, is a bioactive compound that naturally occurs in the plants *Agrimonia pilosa* Ledeb. [1] and *Spiraea formosana* Hayata [2]. AM was first isolated from the fresh root of *A. pilosa* in 1958 by a Japanese scholar [1]. In 2004, AM was subsequently found in the fresh stems of *S*. *formosana* [2].

**Figure 1:**
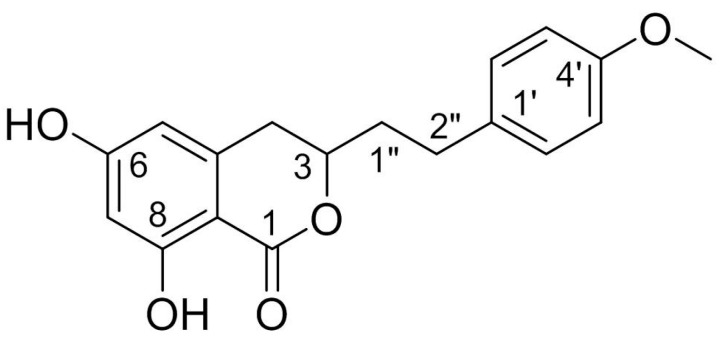
Chemical structure of AM.

Structurally, AM is a derivative of isocoumarins and its molecular formula is C_18_H_18_O_5_. The chemical name of AM is 3,4-dihydro-6,8-dihydroxy-3-[2-(4-methoxyphenyl) ethyl]-1*H*-2-benzopyran-1-one. This racemate compound possesses one methoxyl and two phenolic hydroxyls, one of which is chelated to a lactonic C = O. The chemical structure of AM is shown in Figure 1. 

Investigation of the bioactivities of AM has demonstrated that this compound has a wide range of potential pharmacological activities [3,4,5,6,7,8,9,10,11,12]. According to the current literature, AM acts as an α_1A_ adrenergic receptor antagonist [3]. AM also exerts anti-inflammatory [4], antitumor [5,6], antioxidant [7], hepatoprotective [8], and myocardial protective effects [9,10]. Furthermore, AM effectively increases insulin-mediated glycogen levels in hepatocytes, suggesting that it could be a promising natural product for the prevention and treatment of diabetes mellitus [11,12].

In recent years, there have been several reviews on the botany, phytochemistry, ethnopharmacology, pharmacology, and traditional uses of *A. pilosa* [13,14,15,16], from which AM is isolated. However, there are few systematic summaries of the knowledge about this compound. In particular, important information regarding AM, such as aspects of its in vivo pharmacokinetics, biodistribution, bioavailability, and the application of drug delivery systems, is lacking. These obstacles limit the use and development of AM. 

To promote understanding of the current research status of AM and provide justification for future intensive study and comprehensive application, we provide new insights into AM in this review, focusing on its sources, properties, pharmacological effects, and safety. In addition, we offer an outlook on the future development of this compound, including problematic issues highlighted in previous studies and possible strategies to resolve these. 

## 2. Sources

### 2.1. Derivation from Plants

As mentioned above, AM has been found in the plants of *A*. *pilosa* and *S*. *formosana*, which belong to the Rosaceae family. *A*. *pilosa* is a perennial herb with an erect stem that is 30–120 cm in height, and it grows along roadsides or in grassy areas at diverse altitudes. This plant is distributed in China, central Europe, the former Soviet Union, Mongolia, North Korea, Japan, and northern Vietnam [17]. *A*. *pilosa* is used in traditional Chinese medicine for mainly treating hemoptysis, metrorrhagia, hematemesis, and bloody dysentery [18]. *S*. *formosana* is a shrub endemic to Taiwan that grows in alpine woodlands at an altitude of 2100–2950 m [19]. The tender leaves, fruits, and roots of this plant have traditionally been used as diuretics, antidotes, and analgesics to treat inflammation, cough, headache, and toothache [20,21].

Conventional approaches are usually used to extract and separate AM from the above-mentioned plants. Table 1 summarizes the extraction and separation methods of AM [1,2,4,8,11,22,23]. The extraction and isolation process of AM from *S. formosana* can be summarized as follows [2]. Briefly, the fresh stems of *S. formosana* are extracted with hot ethanol, and the water suspension of the ethanol extract is subjected to a liquid-liquid partition to obtain chloroform, n-butanol, and water subfractions. AM is then separated from the chloroform subfraction using a combination of silica gel column chromatography. Finally, 5.6 mg of AM is obtained from 8.6 kg of *S. formosana*, equivalent to the content of 0.65 mg/kg. This result indicates that the content of AM in *S. formosana* is low.

A large number of reports have described the process of extracting and separating AM from *A. pilosa*. Low toxicity solvents, such as absolute methanol and 60–70% ethanol solutions, are usually used to extract AM. Conventional separation techniques, such as liquid–liquid extraction, silica gel column chromatography, recrystallization, macroporous resin, and preparative high-performance liquid chromatography, have been used to separate AM. AM yields vary from 3–4 mg/kg to 300–400 mg/kg [1,8,11,22]. These classic separation methods are complicated, inefficient, and time consuming. However, in recent years, high-speed counter-current chromatography (HSCCC) coupled with ultraviolet detection or evaporative light-scattering detection has been considered an efficient protocol for separating AM from *A. pilosa* [23]. The AM yield obtained using this method is higher than that using conventional methods, producing an AM content of 770 mg/kg. Therefore, HSCCC is a powerful technique for separating AM from *A. pilosa*.

In summary, there are substantial differences in AM yields extracted from *A. pilosa* owing to differences in the origin of plants, the extraction and separation methods used, extraction parts, and other potential factors. Notably, *A. pilosa* has a much higher content of AM than *S. formosana*, which explains why *A. pilosa* is the major source of AM.

### 2.2. Obtaining AM by Chemical Synthesis

The low accumulation of AM in plants, its cumbersome extraction and separation processes, and the overexploitation of natural resources are generally considered the main driving factors for its high production cost. These factors are also the main causes of supply shortages of AM. These obstacles have made chemical synthesis an appealing alternative method for obtaining AM. Although AM has a variety of pharmacological activities, there have been relatively few advances in its chemical synthesis. 

**Figure 2:**
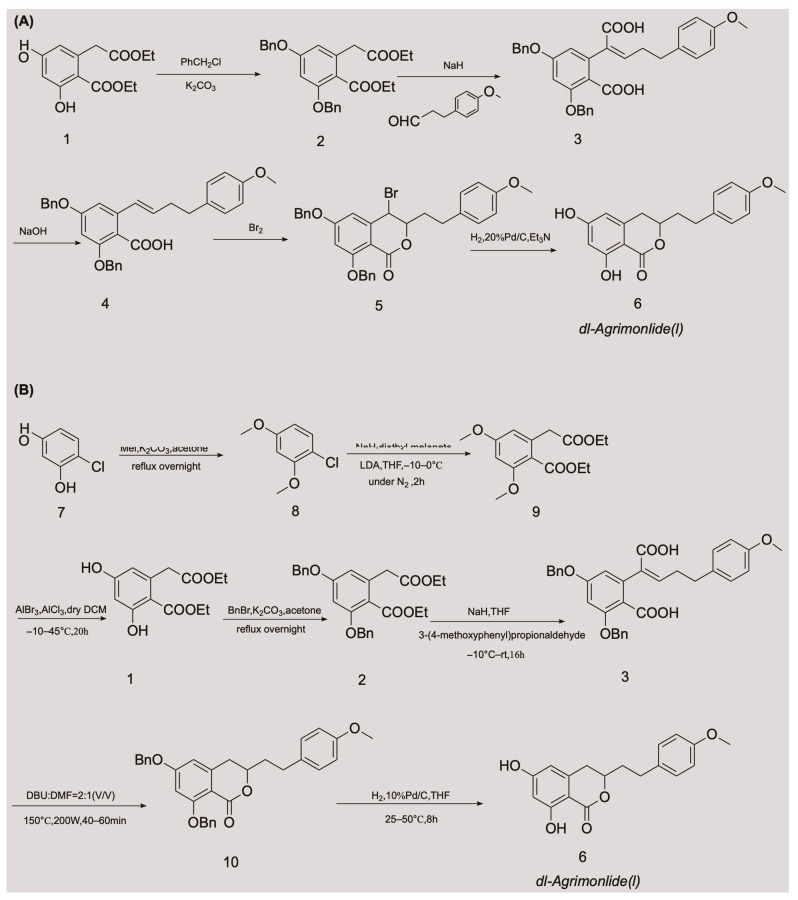
Chemical synthesis routes of AM [24,25,26].

A few attempts have been made to synthesize AM, with variable success. In 1976, Yamato et al. synthesized the racemate of AM in five steps for the first time, and confirmed its structure using nuclear magnetic resonance imaging [24]. They started with compound 1 and obtained compound 2 by protecting two phenolic hydroxyl groups with a benzyl group. An overall yield of 2.6% AM was obtained through a series of reactions, including Stobbe condensation, ester hydrolysis, benzyl decarboxylation, bromine addition, and reduction [24]. The chemical synthesis route is shown in Figure 2A. Unfortunately, there were no subsequent reports of the chemical synthesis process of AM for many years. In 2018, a Chinese invention patent was published that contained a novel chemical synthesis method for AM [25]. The authors improved on the synthesis route of Yamato et al., and constructed the 3,4-dihydroisocoumarin core structure using microwave-assisted intramolecular esterification for the first time. The synthetic process began with 4-chlororesorcinol as the material, and the goal product was obtained in seven steps. The chemical synthesis route is shown in Figure 2B. This synthetic route increased the overall yield of AM to 20.7% [25,26], which is nearly eight times higher than that of Yamato et al. This exciting result suggests that microwave-assisted synthesis is a promising approach for the chemical construction of AM.

## 3. Properties

### 3.1. Physicochemical Properties

Physicochemical properties, such as solubility in water and organic solvents, the acid dissociation constant, the oil/water partition coefficient, and chemical stability, are key factors that affect the pharmacokinetics, biopharmaceutics, and quality of drugs. The physicochemical properties of AM are summarized in detail in Table 2 [27,28,29]. 

AM is a white powder with a molecular weight of 314.3 g/moL [28]. AM is soluble in chloroform, dichloromethane, ethyl acetate, dimethyl sulfoxide, acetone, and other solutions [30], but is only minimally soluble in petroleum ether. This solubility suggests that AM is lipophilic. The partition coefficient and distribution coefficient are important parameters that describe the lipophilicity of a compound, which can be used to help predict the in vivo permeability. The reported partition coefficient and distribution coefficient values of AM are 3.649 and 2.949, respectively [27], indicating that AM has low solubility and moderate permeability. Such compounds usually have good intestinal tract permeability because there is a good balance between dissolution and passive diffusion penetration. According to the biopharmaceutics classification system, drug substances are categorized into four classes on the basis of their solubility parameter and permeability to biomembranes for evaluating the desired results of a formulation on oral bioavailability [31]. A low water solubility and poor oral bioavailability limit the biological effects of many natural products in vivo. The existing data suggest that AM belongs to biopharmaceutics classification system Class II and is likely to exhibit dissolution rate-limited absorption. However, this possibility requires further confirmation by determining the solubility of AM in water.

Regarding chemical stability, contact should be avoided between AM and strong oxidizing agents, reducing agents, strong acids, and alkalis. In the soluble form of AM, it should be sealed and stored below −20 °C to maintain its stability for several months. In the solid form of AM, it is stable at temperatures of 2–8 °C when kept in a dry place [30]. 

### 3.2. Predicted Absorption, Distribution, Metabolism, Excretion, and Toxicity Properties

Pharmacokinetic behaviors of drugs in vivo include absorption, distribution, metabolism, excretion, and toxicity (ADMET). The ADMET properties account for 50% of drug research and development (R&D) failures. Computer-aided design is an effective and alternative method of biological experimental evaluation, and helps to improve the R&D success rate. Computational approaches have increasingly been used to predict ADMET properties of compounds, especially in evaluating the ADMET properties of herbal medicines [32]. The predicted ADMET profiles of AM regarding its absorption, solubility, permeability across the blood-brain barrier (BBB), interactions with cytochrome P450 2D6, hepatotoxicity, and plasma protein binding (PPB) are shown in Table 3 [27].

AM appears to show a good absorption capacity in vivo with a predicted absorption level of 0. However, AM is predicted to have a low aqueous solubility, with a solubility level of 2, which contradicts the prediction result for in vivo absorption and needs to be further confirmed by in vivo testing. Regarding the prediction of BBB penetration, AM exhibits a moderate BBB penetration capability, with a level of 2. This indicates that AM may enter the brain tissue through the BBB and could be used to treat brain diseases. Furthermore, the ADMET predictor shows that AM exhibits potential hepatotoxicity, with a level of 1. Preliminary explorations and in-depth investigations are required to determine the specific mechanism of hepatotoxicity and whether it is dose dependent. In addition, AM is predicted to be a non-inhibitor of the cytochrome P450 2D enzyme and may be metabolized and excreted successfully. Therefore, drug-drug interactions are less likely when AM and the cytochrome P450 2D6 substrates are used simultaneously. Moreover, the PPB level is predicted to be 2, indicating that the binding rate of AM with plasma protein is ≥95%. The high degree of PPB limits the partitioning of AM from the blood into the tissues, where it could be metabolized. This limited partitioning may result in a delayed onset of action and longer half-life period, thereby reducing the elimination of AM. AM has been predicted to have a good drug-likeness, with a drug-likeness weight of 0.842. Generally, AM demonstrates promising ADMET profiles. However, to fully confirm the ADMET properties of AM, real-world tests are required to validate these properties, and more animal and human studies are required.

## 4. Pharmacological Effects

AM possesses a wide range of pharmacological activities, such as antitumor activity, antioxidation and hepatoprotection, antidiabetic activity, anti-inflammatory activity, myocardial protection, and α1A adrenergic receptor antagonist activity. The mechanisms of action of these effects are shown in Table 4.

### 4.1. Antitumor Effects

**Figure 3:**
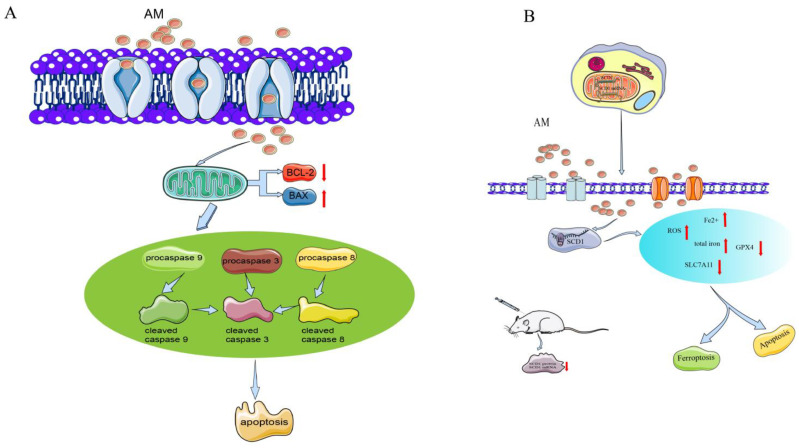
Mechanism of AM for its antitumor effects. (**A**) Anti-gastric cancer; and (**B**) anti-ovarian cancer.

Many studies have shown that *A. pilosa* has good inhibitory effects on a variety of tumors [33,34,35,36]. AM is one of the main active components of *A. pilosa*, and its in vitro antitumor effects have been evaluated in AGS cells of human gastric cancer, in SKOV-3 and A2780 cells of human ovarian cancer [5,6], and in the SKOV-3 xenograft model in vivo [5]. The mechanism of the antitumor effects of AM is shown in Figure 3.

In a study on human gastric cancer AGS cells, AM had a potent antiproliferation activity and dose-dependently enhanced the total number of apoptotic cells [6]. The inhibitory rates at 10, 20, and 40 µM were 30%, 45%, and 67%, respectively. The percentage of cells in the G0/G1 phase was increased by 82.7% and that of the G2/M phase was decreased by 9.0% after treatment with AM at 40 µM. The treatment prevented cell cycle advancement by producing an arrest during the G1 phase. The response of AM to early and late apoptotic cells increased in a dose-dependent manner [6]. 

Furthermore, cleaved caspase activation has been shown to be related to AM-induced cell death. AM downregulates procasapase-3, -8, and -9, and upregulates the active forms of caspase, cleaved caspase-3, -8, and -9. Moreover, the expression of Bax is markedly enhanced, whereas the expression of Bcl-2 appears to be reduced, suggesting the activity of mitochondria in signal transduction during AM-induced apoptosis. AM increases the level of phosphor-extracellular regulated protein kinases (ERKs)/ERK protein and phospho-p38 protein expression, indicating that it affects proliferation and apoptosis through the p38 mitogen-activated protein kinase (MAPK) pathway [6]. 

In A2780 and SKOV-3 cells, AM dose-dependently (10–40 µM) increases the apoptosis rate and the cleavage of caspase-3 and caspase-9, and inhibits the proliferation, migration, and invasion of these cells. AM-induced ferroptosis in ovarian tumor cells elevates the intracellular levels of reactive oxygen species (ROS), total iron, and ferrous iron, and downregulates the concentrations of ferroptosis indicators (SLC7A11 and GPX4 proteins) [5].

Stearoyl-CoA desaturase 1 (SCD1) functions as a lipid-regulating enzyme in the development of human cancer. SCD1 modulates malignant transformation, expedites cancer cell initiation, inhibits cell apoptosis, and promotes cancer cell proliferation in various human cancers [37]. Bioinformatic analysis shows that the binding energy of the interaction is −8.21 kcal/moL, indicating that AM directly targets SCD1 protein. AM can also reduce the growth of ovarian cancer cells and trigger ferroptosis via the control of SCD1 expression in A2780 and SKOV-3 cells [5]. 

In addition, an SKOV-3 xenograft model in vivo has been used to evaluate the activity of AM in ovarian tumors. AM at a dose of 50 mg/kg substantially inhibited tumor volume, size, and weight. The levels of chemical density and protein expression of SCD1 in tumors were substantially reduced [5]. 

The above-mentioned research suggests that AM may be a novel therapeutic agent for treating gastric cancer and ovarian cancer.

### 4.2. Antioxidative and Hepatoprotective Effects

**Figure 4:**
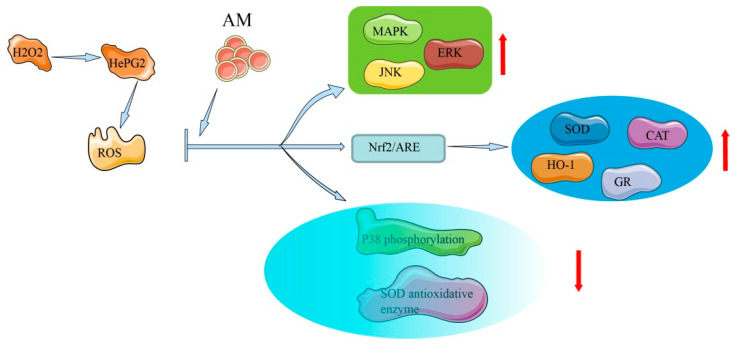
Mechanism of AM for its antioxidative and hepatoprotective effects.

In HepG2 cells and rat primary hepatocytes, AM possesses in vitro hepatoprotective activity that is related to antioxidative effects and free radical eradication [8]. The mechanism of AM in antioxidative and hepatoprotective effects is shown in Figure 4. 

In human liver-derived HepG2 cells, AM exhibits hepatoprotective activity (half-maximal effective concentration [EC_50_] = 88.2 ± 2.8 µM) against tacrine-induced cytotoxicity, demonstrating moderate liver protection compared with silybin (EC_50_ = 69.0 ± 3.4 µM) [8], which is a natural, highly effective, hepatoprotective drug [38]. Exposure of HepG2 cells to AM at a concentration of 200 µM leads to increased heme oxygenase-1 expression via the transcriptional activation of nuclear factor erythroid-2-related factor 2 (Nrf2)/antioxidant response element and inhibition of p38. In oxidative stress, high ROS and electrophile concentrations induce the release of Nrf2 from the cytoplasm and its accumulation in the nucleus, where it encourages the transcription of cytoprotective genes. Through the activation of the antioxidant response element, exposure to oxidative stress triggers a number of antioxidant genes as a defense mechanism, indicating the considerable cytoprotective potential of AM [7]. The mechanisms underlying this protective action include free radical scavenging activities, activation of Nrf2-driven pathways, inhibition of p38 phosphorylation, activation of ERK, c-Jun N-terminal kinase, MAPK phosphorylation, and elevation of the activity of antioxidative enzymes [7,39].

In rat primary hepatocytes, a hepatoprotective effect of AM (EC_50_ = 37.7 ± 1.6 µM) has also been observed and compared with that of silybin (EC_50_ = 67.2 ± 3.5 µM) [8]. The lower EC_50_ value of AM indicates a stronger hepatoprotective action than that of silybin. AM substantially reduces the level of lactate dehydrogenase leakage when 1.5 mM of tert-butyl hydroperoxide is added. Tert-butyl hydroperoxide simulates an oxidative stress condition in the liver and can be metabolized to free radical intermediates [40]. Monitoring oxidative markers among hepatocytes offers the potential to diagnose the extent of liver damage and ultimately to examine the response to medical specialty therapies [41]. The above-mentioned results indicate that the mechanisms underlying AM’s hepatoprotective activity in rats are related to its free radical scavenging activity. 

### 4.3. Antidiabetic Potential

**Figure 5:**
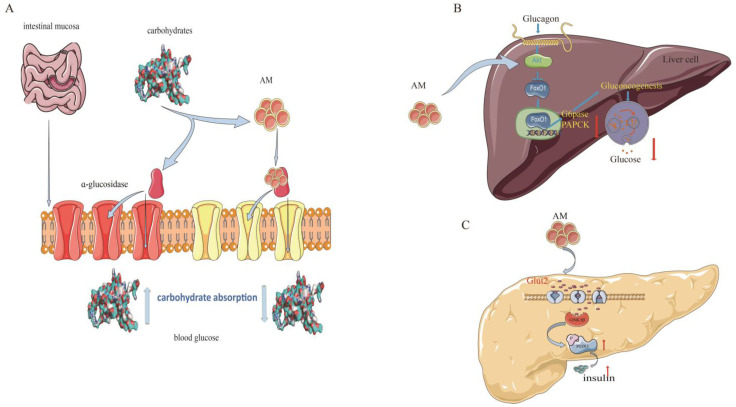
Mechanism of AM for its anti-diabetic effects. (**A**) Inhibition of α-glucosidase; (**B**) constraint of gluconeogenesis; and (**C**) promotion of pancreatic duodenal homeobox-1 expression.

Type 2 diabetes mellitus (T2DM) is the most common type of diabetes and accounts for more than 90% of all diabetes cases [42]. In recent years, scientific research has supported the use of AM for the prevention and treatment of T2DM. The mechanisms of AM against T2DM are related to its regulation of sugar metabolism-related enzymes and insulin signaling pathways [11,12,22] (Figure 5).

Alpha-glucosidase is a critical enzyme associated with T2DM. The inhibition of α-glucosidase is considered an effective approach or treatment for T2DM owing to its potential for reducing glucose absorption by preventing carbohydrate digestion [43]. AM shows a strong α-glucosidase inhibitory activity, with a half-maximal inhibitory concentration (IC_50_) value of 37.4 µM and noncompetitive inhibition, with a Michaelis constant (k_I_) of 17.3 µM, which is higher than that of the representative α-glucosidase inhibitor acarbose (IC_50_ = 45.2 µM, k_I_ = 22.5 µM) [11]. 

Glucokinase (GK), glucose-6-phosphatase (G6Pase), and phosphoenolpyruvate carboxykinase (PEPCK) are other important regulators in diabetes. Balancing the fluxes through GK and G6Pase determines the production of hepatic glucose [12]. PEPCK is a member of the lyase family and is active in the metabolic pathway of gluconeogenesis [44]. AM has shown a potential to regulate glucose metabolism in insulin-resistant HepG2 cells. The ability of AM to increase insulin-mediated glycogen levels in hepatocytes is comparable to that of metformin. At a concentration of 20 µM, AM substantially elevates (*p* < 0.05) GK activity (3.0 U/min/mg protein). AM also substantially reduces G6Pase and PEPCK activities [12]. 

Pancreatic duodenal homeobox-1 (PDX-1) is currently recognized as a specific marker of pancreatic stem cells. PDX-1 regulates the development of the pancreas, promotes pancreatic islet β-cell differentiation, and maintains mature islet β-cell functions. The reduction in PDX-1 activity may be a critical mediator that causes dysregulation of pancreatic β-cells in T2DM [45]. In a dual luciferase reporter gene assay test, AM at a concentration of 1 µmol/L had a facilitatory effect on PDX-1 expression, with a rate of 22.9% (*p* < 0.01). This effect was further confirmed using western blotting, which showed that AM at a concentration of 5 µmol/L promoted the expression of PDX-1 [22]. These findings suggest that AM is a promising natural product for diabetes treatments.

### 4.4. Anti-Inflammatory Effects

Inflammation is a defensive response of the body to stimuli and is a complex process caused by many factors. Most chronic diseases caused by lifestyle factors appear to be related to inflammation [46]. AM has a strong anti-inflammatory activity and may play an important role in the prevention and alleviation of inflammatory diseases [4]. In lipopolysaccharide-stimulated RAW 264.7 cell models, AM at a concentration of 80 µM substantially reduced mRNA expression of proinflammatory cytokines, such as interleukin (IL)-6 and tumor necrosis factor-α. Nitric oxide (NO) production was also inhibited by treatment with AM in a dose-dependent manner (20–80 µM). At a concentration of 80 µM, AM exhibited strong NO inhibitory activity with a rate of 85.36%. Moreover, AM substantially and dose-dependently decreased the production and mRNA expression of cyclooxygenase-2 (COX-2) and nitric oxide synthase (iNOS). After treatment with AM at 80 µM, the expression levels of iNOS and COX-2 protein in macrophages were substantially decreased by 78% and 61%, respectively [4]. 

**Figure 6:**
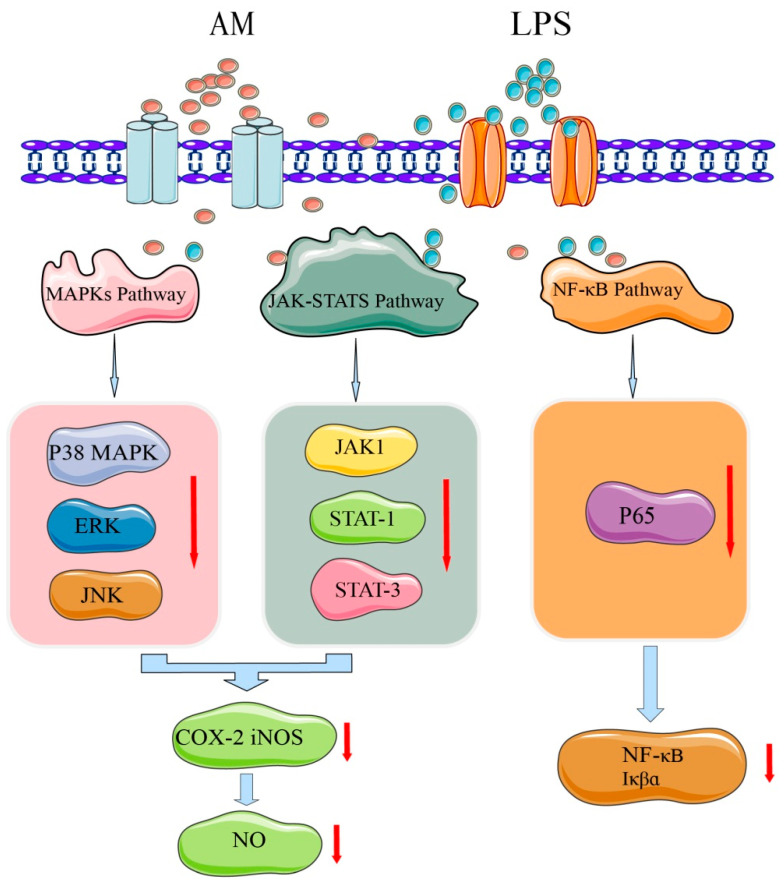
Mechanism of AM for its anti-inflammatory effects.

To identify the potential mechanisms of AM at the molecular level and explain its anti-inflammatory effect, the activation mechanisms of nuclear factor (NF)-κB, MAPK, and Janus kinase signal transducer and activator of transcription (JAK-STAT) signaling pathways have been further investigated [4]. The mechanism underlying AM’s anti-inflammatory effect is shown in Figure 6. NF-κB is an important mediator of the mechanical activation of inflammation [46]. AM at 40–80 µM was found to substantially inhibit the DNA binding activity of NF-κB p65 and the phosphorylation of IκBα [4]. MAPKs regulate lipopolysaccharide-induced inflammatory and immune responses of macrophages [47]. Treatment with 80 µM of AM may substantially downregulate the activation of MAPKs, including c-Jun N-terminal kinase, ERK, and p38 kinase, which are involved in inflammation [4]. JAK-STATs are other signaling pathways that induce the expression of various critical mediators of inflammation [48]. AM at 80 µM substantially blocks the phosphorylation of JAK1, STAT1, and STAT3 [4]. These findings indicate that the anti-inflammatory activity of AM may suppress the activation of the JAK-STAT and p38 MAPK signaling pathways. 

### 4.5. Myocardial Protective Effects

**Figure 7:**
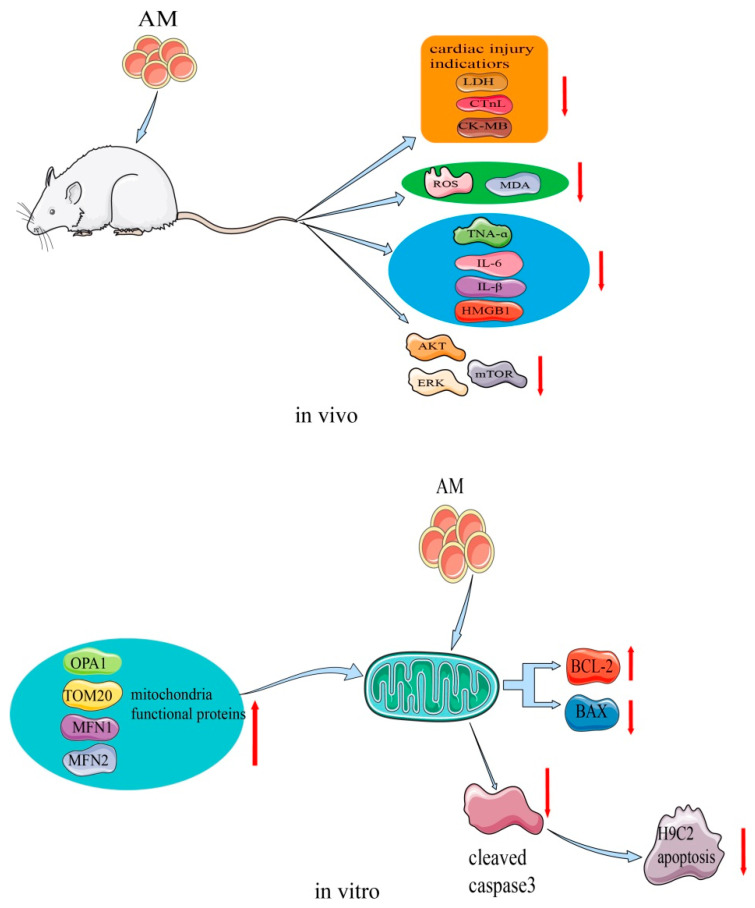
Mechanism of AM for its myocardial protective effects.

AM plays an important role in the process of restoring myocardial damage. This effect has been confirmed in H9c2 cells and a cecal ligation and puncture (CLP) rat model [9,10]. The mechanism of the myocardial protective effects of AM is shown in Figure 7. A study showed that, at a concentration of 15 µM, AM stimulated H9c2 cell proliferation and increased cellular adenosine triphosphate content [10]. In the transcriptome sequencing test, AM caused altered expression of genes that are mainly involved in the mitochondrial function of H9c2 cells. By regulating the expression of apoptosis-related proteins, AM decreased the levels of cleaved caspase 3 and Bax, and elevated Bcl2 levels, thus preventing the rate of apoptosis and shielding H9c2 cells from hypoxia-induced apoptosis. Additionally, hypoxia-stimulated ROS production was markedly reduced by the same concentration of AM. Transmission electron microscopy further suggested that AM reduced the appearance of vacuoles, prevented the disappearance of the mitochondrial crest, and prevented damage to the mitochondrial membrane structure, thereby preserving the normal shape of mitochondria. The expression of mitochondrial functional proteins, such as OPA1, MFN1, MFN2, and Tom20, was increased. These findings indicate that AM regulates the functional proteins in mitochondria to enhance mitochondrial activity. 

Additionally, in the CLP rat model, AM at a dose of 5 mg/kg attenuated sepsis-induced myocardial injury by affecting Akt signaling [9]. The levels of several cardiac injury indicators, such as lactate dehydrogenase, cardiac troponin, and creatine kinase-MB, were substantially reduced by AM, indicating that it may attenuate CLP-induced myocardial injury in vivo. Oxidative stress in CLP rats decreased with AM treatment, and ROS and malondialdehyde levels also decreased to nearly normal. AM showed strong potential inhibition of inflammation by greatly decreasing the levels of tumor necrosis factor-α, IL-6, IL-β, and high mobility group box 1. Moreover, AM strongly suppressed the activation of Akt, ERK, mammalian target of rapamycin, and the apoptosis of cardiomyocytes induced in CLP rats [9]. 

### 4.6. Blocking the α_1A_ Adrenergic Receptor

The α_1A_ adrenergic receptor is a member of the G-protein-coupled receptor family, which mediates the signal transduction of cell membranes. This signal transduction increases the levels of secondary messengers and leads to the release of a large amount of sodium ions into the endoplasmic reticulum to induce smooth muscle contraction [49]. This receptor is abundant in the prostate of Sprague-Dawley rats [50]. The effects of AM on α1A adrenergic receptors have been recognized, analyzed, and identified using Sprague-Dawley rat prostate cell membrane chromatography-online coupled with high-performance liquid chromatography/mass spectrometry [3]. These results provided preliminary evidence that AM is a potential α_1A_ adrenergic receptor antagonist, which may be useful for preventing chronic prostatitis. However, the relevant mechanisms of this effect remain to be clarified and require further exploration.

## 5. Safety

To the best of our knowledge, the available data on the safety of AM are limited. Safety studies on AM have mainly focused on in vitro 3-(4,5-dimethylthiazol-2-yl)-2,5-diphenyltetrazolium bromide (MTT) tests of different types of cells, and AM shows no cytotoxicity over a range of concentrations [4,5,6,7,8,12]. Various concentrations of AM (25–200 µM) do not cause a substantial change in viability, or in the size and shape of HepG2 cells. Even at a high concentration of 200 µM, no cell shrinkage or shedding of adhesion molecules occurs [7]. In a hepatoprotective activity study, the survival rate of HepG2 cells was not altered in the presence (1–100 µM) or absence of AM [8]. A similar result was also found for HepG2 cells treated with AM at a concentration of 20 µM [12]. In a study of cytotoxic effects on RAW 264.7 cells, AM concentrations of 20–80 µM did not cause any changes in MTT-based cell viability. Annexin V/propidium iodide staining has been further used to characterize the cytotoxic effect of AM. After treatment with AM, the proportion of early apoptotic cells substantially decreases. These results indicate that AM does not show signs of cytotoxicity in RAW 264.7 cells, and that it does not inhibit the early events leading to apoptosis [4]. In another MTT test, AM concentrations of 10–40 µM showed no cytotoxicity on the growth of HepG2, HT-29, or MCF-7 cells [6]. The above-mentioned cytotoxicity tests provide preliminarily support for the safety of AM in vitro.

## 6. Conclusions and Future Perspectives

This review has provided deep insight into the sources, properties, pharmacological effects, and safety of AM, a promising bioactive compound from *A. pilosa*. AM acts as a potential agent against cancer, inflammation, hepatic injury, myocardial damage, and diabetes mellitus. The possible mechanisms of its pharmacological effects have been clarified at cellular and molecular levels. Furthermore, AM shows no cytotoxicity over a range of concentrations in different types of cells, providing evidence for its good safety profile in vitro. In addition, the ADMET prediction of AM shows a moderate BBB penetration ability, a long half-life, and good drug-likeness. These results prompt that AM has potential application prospects in medical and pharmaceutical industries. 

Despite the many encouraging achievements mentioned above, there is still much scope to improve the R&D of AM. AM was discovered as early as the 1950s. However, AM has surprisingly not yet received widespread attention, as reflected in the limited number of relevant publications and relatively scattered research foci. Moreover, the existing research lacks depth and systematicity, and research in some areas, such as pharmacokinetics, metabolism, and disposition, is lacking. The following aspects of AM research could be improved, with particular attention being paid to the areas in which research is lacking. 

The content of AM in *A. pilosa* is not high, and the HSCCC separation method produces a yield of only 770 mg/kg. Therefore, the development of an efficient, environmentally friendly, and simple chemical synthesis process is urgently required. Natural products generally have a distinctive metabolism and a structure that is too complex for profitable production by total chemical synthesis [51]. Biosynthesis, which is a newly developed benchmark strategy to reach industrial-scale production, is a new application trend that could increase the production of AM [51]. Additionally, current information on the chemical stability of AM is insufficient. Because of the lactone grouping of AM, it is likely to undergo a hydrolysis reaction, which may be affected by temperature, pH, ionic strength, and other factors. However, the molecular structure of AM contains phenolic hydroxyl groups and is prone to oxidation. The specific conditions of hydrolysis and oxidation of AM require further confirmation using systematic stress tests. With regard to the pharmacological effects of AM, most previous studies were in vitro cell experiments. Therefore, the exploration of AM mechanisms is insufficient. In the next stage of research, relevant animal experiments should be performed to determine the mechanisms of action of AM in as much detail as possible. The literature shows that at least 20 different types of pharmacological activities have been reported for *A. pilosa.* Therefore, AM may possess more pharmacological effects than those already documented. Further research is required to discover new pharmacological activities and mechanisms of AM. Furthermore, although all of the ADMET profiles of AM have been predicted by computing software, they have not been confirmed by real-world experiments; therefore, their reliability is unknown. Specific in vitro or in vivo ADMET studies are required to promote understanding of the pharmacokinetics, metabolism, and disposition of AM. More importantly, future applications of AM will depend on the quality of the design of drug delivery systems and corresponding preparation strategies targeted to specific functions. This application will require new pre-formulation and formulation developments. Finally, but most importantly, substantial work is required regarding the safety of AM. In addition to cytotoxicity tests, comprehensive and systematic safety evaluations (e.g., for acute toxicity, subacute toxicity, cumulative toxicity, genetic toxicity, and hepatotoxicity) should be performed. Importantly, as mentioned in the in silico ADMET predictions, AM may have hepatotoxic potential. This potential needs to be confirmed by sufficient and strong evidence from in vivo experiments. If AM is hepatotoxic, the relationship between dose and toxicity, and how AM affects the liver, must be clarified. In summary, a thorough understanding of AM from all perspectives would help to accelerate its development, application, and clinical transformation.

## Figures and Tables

**Table 1:** Extraction and separation methods of AM.

Parts	Methods of Extraction and Isolation	Yield	Content	Ref.
Fresh stems	8.6 kg of *S. formosana* is extracted with hot ethanol, and the water suspension of the ethanol extract is subjected to a liquid-liquid partition to obtain chloroform, n-butanol, and water subfractions, respectively. The chloroform subfraction is then fractionated by silica gel column chromatography.	5.6 mg	0.65 mg/kg	[2]
Fresh roots	10 kg of *A. pilosa* is extracted with methanol, and the extract is shaken with diethyl ether. The soluble part is boiled several times with petroleum ether, and the residue is heated and extracted repeatedly with benzene. Finally, the precipitated crystals are recrystallized from benzene and then from methanol.	3000–4000 mg	300–400 mg/kg	[1]
Dried plant	50 kg of *A. pilosa* is extracted with 60% ethanol, and the 30% ethanol elution part of macroporous resin is separated by silica gel column chromatography, recrystallization, ODS column chromatography, Sephadex LH-20 gel column chromatography and preparative high-performance liquid chromatography.	202 mg	4.04 mg/kg	[22]
Dried aerial parts	13 kg of *A. pilosa* is extracted with methanol and the extract is suspended in water. The suspension is partitioned between hexane, ethyl acetate, and n-butanol. The ethyl acetate fraction is then fractionated by repeated silica gel column chromatography.	43.7 mg	3.36 mg/kg	[11]
NA	Ethyl acetate fraction of methanol extract of *A. pilosa* is chromatographed repeatedly with silica gel columns and purified by preparative thin layer chromatography.	6.5 mg	NA	[4]
Dried roots	290 g of *A. pilosa* is extracted with hot water and the filtrated aqueous solution is partitioned with ethyl acetate and n-butanol, successively. The ethyl acetate soluble fraction is chromatographed by silica gel column repeatedly.	44 mg	151.7 mg/kg	[8]
Dried plant	500 g of *A. pilosa* is extracted with 70% ethanol. The extract is then eluted with different concentrations of ethanol on the macroporous resin. The 50% ethanol eluted fractions is collected and used for subsequent high-speed counter-current chromatography separation.	385.2 mg	770.4 mg/kg	[23]

NA: not available.

**Table 2:** Physicochemical properties of AM.

Physicochemical Properties	Property Value	Ref.
color/form	white powder	[28]
molecular weight	314.3 g/moL	[28]
partition coefficient	3.649	[27]
distribution coefficient	2.949	[27]
acid dissociation constant	8.10 ± 0.40	[28]
density	1.293 g/cm3	[28]
melting point	175.5–176.5 °C	[29]
boiling point	581.1 °C at 760 mmHg	[28]
refractive index	1.611	[28]
flash point	215.5 °C	[28]
vapour pressure	4.2E–14 mmHg at 25 °C	[28]

**Table 3:** Predicted ADMET properties of AM [27].

ADMET Properties	Prediction Value	Level
ADMET absorption	/	0
ADMET BBB	–0.241	2
ADMET solubility	–4.092	2
ADMET hepatotoxicity	0.655	1
ADMET CYP2D6	0.356	0
ADMET PPB	/	2
drug-likeness	0.842	good

ADMET absorption levels: 0, 1, 2, and 3 represent good, moderate, low, or very low absorption, respectively. ADMET BBB levels: 0, 1, 2, 3, 4, and 5 represent very high, high, medium, low, undefined, and molecules with one or more unknown AlogP98 types, respectively. ADMET solubility levels: 0, 1, 2, 3, 4, 5, and 6 represent extremely low, very low but possible, low, good, optimal, too soluble, and molecules with one or more unknown AlogP98 types, respectively. ADMET hepatotoxicity: 0 and 1 represent nontoxic and toxic effects, respectively. ADMET CYP2D6: 0 and 1 represent non-inhibitor and inhibitor, respectively. ADMET PPB levels: 0, 1, and 2 represent binding <90%, binding ≥90% and binding ≥95%, respectively.

**Table 4:** Mechanisms of pharmacological effects of AM.

Pharmacological Effects	Levels	Models	Concentrations or Doses of AM	Mechanisms	Ref.
anti-gastric cancer	in vitro	AGS cells	40 µM, IC_50_ = 25.9 μM	decrease the expression of Bcl-2; increase the expression of Bax; increase the level of phospho-ERK/ERK protein and the expression of phosphor-p38 protein; increase the activity of caspase-3; down-regulate the levels of the inactive pro-caspase-3, -8, and -9 proteins	[6]
anti-ovarian cancer	in vitro	A2780 and SKOV-3 cells	40 µM	increase the cleavage of caspase-3 and -9; increase the levels of ROS, total iron and ferrous ion, and down-regulate the levels of SLC7A11 and GPX4, thus inducing ferroptosis; direct inhibit tumor cell migration and invasion; inhibit the protein levels of SCD1	[5]
	in vivo	SKOV-3 xenograft model (BALB/c mice)	50 mg/kg	down-regulate the expressions of Ki-67 and SCD1; lower the expressions of SCD1 mRNA and protein	[5]
anti-diabetic	in vitro	PANC-1 cell	1 μM; 5 μM	promote the expression of PDX-1	[22]
	in vitro	/	IC_50_ = 37.4 μM	inhibit α-glucosidase	[11]
	in vitro	Insulin-resistance HepG2 cell	20 µM	elevate the activity of GK, and increase the content of glycogen; lower the activities of PEPCK and G6Pase, and constrain the gluconeogensis	[12]
anti-oxidative and hepatoprotective	in vitro	HepG2 cell; rat primary hepatocytes	EC_50_ = 88.2 μM; EC_50_ = 37.7 μM	scavenge the free radical	[8]
	in vitro	HepG2 cell	200 μM	scavenge the free radical; activate Nrf2-driven pathways; activate ERK, JNK, and MAPK phosphorylation; inhibit p38 phosphorylation; elevate the activity of antioxidative enzymes	[7]
anti-inflammatory	in vitro	RAW 264.7 cells	80 μM	reduce the levels of IL-1β, IL-6, and TNF-α; attenuate the expression of iNOS and COX-2; inhibit the activation of JNK and p38 MAPKs; decrease the activation of JAK-STAT and NF-κB	[4]
myocardial protective	in vitro	H9c2 cell	15 μM	regulate the gene expression involved in mitochondrial function; decrease the levels of cleaved Caspase 3 and Bax; boost the level of Bcl2; prevent the rate of apoptosis and shield H9c2 cells from hypoxia-induced apoptosis; reduce ROS production and preserve the normal shape of mitochondria; regulate the functional proteins to enhance the mitochondrial activity	[10]
	in vivo	CLP rat model	5 mg/kg	attenuate myocardial injury by Akt signaling; suppress cardiac injury indicators, oxidative stress, and inflammation; restrain the activation of Akt, Erk, mTOR and the apoptosis of cardiomyocytes	[9]
blocking α_1A_ adrenergic receptor	in vitro	rat prostate cell membrane	/	/	[3]

## Data Availability

No data were used for the research described in the paper.

## Abbreviations

AM, agrimonolide; HSCCC, high-speed counter-current chromatography; ADMET, absorption, distribution, metabolism, excretion, and toxicity; R&D, research and development; BBB, blood–brain barrier; PPB, plasma protein binding; ERK, extracellular regulated protein kinase; MAPK, mitogen-activated protein kinase; ROS, reactive oxygen species; SCD1, stearoyl-CoA desaturase 1; EC_50_, half-maximal effective concentration; Nrf2, nuclear factor erythroid-2-related factor 2; T2DM, type 2 diabetes mellitus; IC_50_, half-maximal inhibitory concentration; GK, glucokinase; G6Pase, glucose-6-phosphatase; PEPCK, phosphoenolpyruvate carboxykinase; PDX-1, pancreatic duodenal homeobox-1; NO, nitric oxide; COX-2, cyclooxygenase-2; iNOS, nitric oxide synthase; NF, nuclear factor; JAK-STAT, Janus kinase signal transducer and activator of transcription; CLP, cecal ligation and puncture; MTT, 3-(4,5-dimethylthiazol-2-yl)-2,5-diphenyltetrazolium bromide.
